# *Mek1* and *Mek2* Functional Redundancy in Erythropoiesis

**DOI:** 10.3389/fcell.2021.639022

**Published:** 2021-07-27

**Authors:** Laurent Beuret, Simon-Pierre Fortier-Beaulieu, Vincent Rondeau, Sophie Roy, Nicolas Houde, Karl Balabanian, Marion Espéli, Jean Charron

**Affiliations:** ^1^Centre de Recherche sur le Cancer de l’Université Laval, Centre de Recherche du CHU de Québec-Université Laval (Oncology), Québec, QC, Canada; ^2^Université de Paris, Institut de Recherche Saint Louis, EMiLy, Inserm U1160, Paris, France; ^3^OPALE Carnot Institute, The Organization for Partnerships in Leukemia, Hôpital Saint-Louis, Paris, France; ^4^Department of Molecular Biology, Medical Biochemistry and Pathology, Université Laval, Québec, QC, Canada

**Keywords:** ERK/MAP kinase pathway, *Mek* genes, hematopoiesis, erythropoiesis, gene inactivation

## Abstract

Several studies have established the crucial role of the extracellular signal–regulated kinase (ERK)/mitogen-activated protein kinase pathway in hematopoietic cell proliferation and differentiation. MEK1 and MEK2 phosphorylate and activate ERK1 and ERK2. However, whether MEK1 and MEK2 differentially regulate these processes is unknown. To define the function of *Mek* genes in the activation of the ERK pathway during hematopoiesis, we generated a mutant mouse line carrying a hematopoietic-specific deletion of the *Mek1* gene function in a *Mek2* null background. Inactivation of both *Mek1* and *Mek2* genes resulted in death shortly after birth with a severe anemia revealing the essential role of the ERK pathway in erythropoiesis. *Mek1* and *Mek2* functional ablation also affected lymphopoiesis and myelopoiesis. In contrast, mice that retained one functional *Mek1* (1*Mek1*) or *Mek2* (1*Mek2*) allele in hematopoietic cells were viable and fertile. 1*Mek1* and 1*Mek2* mutants showed mild signs of anemia and splenomegaly, but the half-life of their red blood cells and the response to erythropoietic stress were not altered, suggesting a certain level of *Mek* redundancy for sustaining functional erythropoiesis. However, subtle differences in multipotent progenitor distribution in the bone marrow were observed in 1*Mek1* mice, suggesting that the two *Mek* genes might differentially regulate early hematopoiesis.

## Introduction

Mitogen-activated protein kinase (MAPK) signaling pathways consist of sequences of protein kinases that link extracellular stimuli to targets located in different cellular compartments (Wortzel and Seger, 2011). In mammals, the extracellular signal–regulated kinase (ERK) cascade constitutes one of the best-characterized MAPK pathways. It is activated by multiple surface receptors such as growth factor receptors and G protein–coupled receptors to elicit various physiological outputs including fate determination, differentiation, proliferation, survival, and migration (Alberola-Ila and Hernandez-Hoyos, 2003; Fischer et al., 2005; Shaul and Seger, 2007; Geest and Coffer, 2009; Guihard et al., 2010; Wortzel and Seger, 2011; Saulnier et al., 2012; Sun et al., 2015). Following receptor triggering, RAS binds GTP and with the help of scaffolding proteins forms heterodimers with RAF protein kinases that directly phosphorylate and activate MEK1 and MEK2, two dual-specificity kinases that activate ERK1 and ERK2 by phosphorylation (Roskoski, 2012a,b). Although multiple kinases can activate MEK1 and MEK2, they are the only kinases able to phosphorylate ERK1 and ERK2, acting as gatekeepers. As such, MEK proteins have been considered potential therapeutic targets as overactivation of ERK pathway is observed in many cancers (Zhao and Adjei, 2014; Caunt et al., 2015).

The ubiquitously expressed MEK1 and MEK2 proteins share a high level of amino acid sequence homology (Aoidi et al., 2016). *Mek1* null mutant mice die during gestation from the underdevelopment of the placenta, whereas *Mek2* null mutants are viable and fertile revealing compensatory effects by *Mek1* in extraembryonic tissues (Bélanger et al., 2003; Bissonauth et al., 2006; Nadeau et al., 2009; Nadeau and Charron, 2014). However, when the deletion of *Mek1* is restricted to the embryo proper, *Mek1* null mice are viable and fertile, indicating functional redundancy between *Mek1* and *Mek2* genes in embryo formation (Bissonauth et al., 2006). Moreover, knock-in of *Mek2* cDNA sequence into the *Mek1* locus rescues the placental defects in absence of MEK1 revealing MEK redundancy, as well as the importance of the total amount of MEK proteins in placenta development (Aoidi et al., 2016). Functional redundancy between *Mek1* and *Mek2* is further supported by the lack of phenotypes in animals in which targeted tissues retain either one *Mek1* or one *Mek2* functional allele (Scholl et al., 2007; Newbern et al., 2008; Yamashita et al., 2011; Li et al., 2012; Shim et al., 2013; Boucherat et al., 2014; Ihermann-Hella et al., 2014; Boucherat et al., 2017). Except for the formation of extraembryonic structures, obvious developmental defects are observed only when all *Mek1* and *Mek2* alleles are deleted.

Several studies have established the crucial role of the ERK pathway in hematopoietic cell proliferation and differentiation (Alberola-Ila and Hernandez-Hoyos, 2003; Fischer et al., 2005; Geest and Coffer, 2009; Guihard et al., 2010; Saulnier et al., 2012; Kollmann et al., 2017). *In vitro* studies using differentiation-competent cell lines showed that the pathway is important for erythroid, megakaryocyte, and myeloid cell growth, apoptosis, and differentiation (Matsuzaki et al., 2000; Uchida et al., 2001; Mori et al., 2003; Geest et al., 2009). The ERK signaling pathway was also shown to control early myeloid commitment of hematopoietic stem cells (HSCs) (Hsu et al., 2007). Moreover, *in vivo* analyses supported a role for the pathway in hematopoiesis. For instance, characterization of *B-raf* null mutant mice revealed a role for the ERK pathway in the expansion of progenitors but not in their differentiation (Kamata et al., 2005). *Erk1* null mutant animals presented accumulation of erythroid progenitors in enlarged spleen indicating that ERK1 may act as a negative regulator of adult steady-state splenic erythropoiesis (Guihard et al., 2010). In bone marrow, *Erk1* deficiency caused mild osteopetrosis due to defective myeloid lineage progenitors and subsequent impairment in osteoclastogenesis and osteoclast function (Saulnier et al., 2012). Altogether, *in vitro* and *in vivo* studies demonstrated that the integrity of the ERK pathway is critical for both erythroid and myeloid cell fate and differentiation. However, while the implication of the ERK pathway in hematopoiesis is established, the function of each kinase of the pathway remains undefined.

To specifically address the role of the ERK activators, MEK1 and MEK2, in hematopoietic cell differentiation, and to bypass the early embryonic lethality associated with the *Mek1* null mutation, we specifically deleted the *Mek1* gene in hematopoietic cell lineages using the *Vav1-icre* deleter mouse line. This conditional mutation was implemented in a *Mek2* null background. Mice lacking *Mek1* and *Mek2* gene functions in the hematopoietic lineages died shortly after birth from anemia. In contrast, mice that retained one functional allele of *Mek1* (1*Mek1*) or of *Mek2* (1*Mek2*) in these lineages were viable and fertile. Both 1*Mek1* and 1*Mek2* mutant mice showed a splenomegaly with reduction of hematocrit, red blood cell (RBC) counts, and hemoglobin concentration in circulating blood, indicating a trend to anemia. Multipotent hematopoietic progenitors were reduced in 1*Mek1* bone marrow but not in spleen. Conversely, multipotent hematopoietic progenitors and erythroid progenitors were significantly increased in 1*Mek2* spleen. Together, these data confirmed the importance of the ERK pathway in erythropoiesis. Despite *Mek1* and *Mek2* gene redundancy in this process, each locus possesses some functional specificity.

## Materials and Methods

### Mouse Strains, Genotyping, and Tissue Collection

The *Mek1*^*flox/flox*^ and *Mek2*^–/–^ mutant mouse lines and the *Sox2-cre* and *Vav1-icre* deleter mouse lines were previously described (Hayashi et al., 2002; Bélanger et al., 2003; de Boer et al., 2003; Bissonauth et al., 2006). *Mek1*^–/–^ mice (*Mek1*^*flox/flox*^ *Tg*^+/Sox2–*cre*^) were obtained by breeding *Mek1*^*flox/flox*^ *Tg*^+/Sox2–*cre*^ males with *Mek1*^*flox/flox*^ females. The 1*Mek1* (*Mek1*^+/flox^ *Mek2*^–/–^ *Tg*^+/Vav1–*iCre*^) mice were generated by breeding *Mek1*^*flox/flox*^ *Mek2*^–/–^ females with 1*Mek1* males, whereas 1*Mek2* (*Mek1*^*flox/flox*^ *Mek2*^+/–^ *Tg*^+/Vav1–*iCre*^) mice were produced by mating *Mek1*^*flox/flox*^ *Mek2*^–/–^ females with 1*Mek2* males. All mouse lines were maintained in the 129S6/SvEv genetic background. Age of the embryos was estimated by considering the morning of the day of the vaginal plug as E0.5. Genomic DNA from embryonic yolk sac and mouse tail biopsy was extracted, purified, and genotyped by Southern blot and polymerase chain reaction analyses as previously described (de Boer et al., 2003; Nadeau et al., 2009). Experiments were performed according to the guidelines of the Canadian Council on Animal Care and in compliance with the European Union guide for the care and use of laboratory animals and approved by the institutional animal care committee and by an institutional review committee (C2EA-26, Animal Care and Use Committee, Villejuif, France).

### Hematologic Parameters

Blood cell counts were analyzed with a UniCel DxC 600 Synchron Clinical Systems (Beckman Coulter, Mississauga, On, Canada). Hematocrit was assessed on a hematocrit centrifuge (IEC Micro MB centrifuge, Saint-Laurent, QC, Canada). Reticulocyte counts were measured as described (Guihard et al., 2010). Briefly, 2 μL of whole blood was incubated with thiazole orange at 10^–4^ mg/mL in phosphate-buffered saline (PBS) for 1 h. Blood sample diluted in PBS was used as an unstained control. Reticulocyte counts were measured by flow cytometry.

### Flow Cytometry

Single-cell suspensions were prepared from mouse hematopoietic tissues by sieving and gentle pipetting through 70-μm nylon mesh. Flow cytometry was used to assess cell populations. Peripheral blood cells and single-cell suspensions (10^6^) were saturated with Fc Block and stained with fluorochrome-conjugated antibodies using standard procedures (Malynn et al., 1995; Bélanger et al., 2003; Espeli et al., 2011). For intracellular staining, cells were fixed 15 min in 2% paraformaldehyde (PFA) at room temperature after labeling with cell surface markers followed by cell permeabilization in ice-cold methanol. Flow cytometry was performed on a BD LSRFortessa cell analyzer. FlowJo software was used for analysis of flow cytometry data using the following mAbs: anti–mouse CD3 [clone 145-2C11, hamster immunoglobulin G1 (IgG1)], CD11b (clone M1/70, rat IgG2b), CD16/32 (clone 93, rat IgG2a), CD34 (clone RAM34, rat IgG2a), CD41 (clone MWReg30, rat IgG1), CD45R/B220 (clone RA3-6B2, rat IgG2a), CD48 (clone HM48-1, Armenian hamster IgG), CD71 (clone RI7217, rat monoclonal), CD117/c-Kit (clone 2B8, rat IgG2b), CD127 (clone A7R34, rat IgG2a), CD135/Flt3 (clone A2F10, rat IgG2a), CD150 (clone TC15-23F12,2, rat IgG2a), Ter119 (clone TER-119, rat IgG2b), Gr-1 (clone RB6-8C5, rat IgG2b), Sca-1 (clone E13-161,7, rat IgG2a), and MEK1/2 (clone D1A5, rabbit IgG). Antibodies were conjugated to biotin, BV650, fluorescein isothiocyanate, phycoerythrin (PE), allophycocyanin (APC), AF700, PE-cyanin (Cy) 5, PE-Cy7, eFluor 450,AF647,APC- eFluor780, peridinin chlorophyll protein PerCP-Cy5,5, or Pacific blue and purchased from BD (Mississauga, ON, Canada), eBioscience (San Diego, CA, United States), or BioLegend (San Diego, CA, United States).

### Colony-Forming Assays

Methylcellulose colony-forming assays were performed in MethoCult M3434 complete medium with recombinant cytokines (StemCell Technologies, Vancouver, BC, Canada). In brief, 2 × 10^4^ cells from bone marrow, 1 × 10^5^ cells from spleen, 2 × 10^4^ peripheral blood cells from E12.5 embryos, 2 × 10^4^ fetal liver cells from E14.5 embryos, and the aorta–gonad–mesonephros (AGM) region from E8.5 embryos were plated for colony-forming unit (CFU) assays. Cultures were incubated at 37°C in 5% CO_2_ for 7 days for the growth of colonies of erythroid burst-forming units (BFU-E); granulocyte, erythrocyte, macrophage, and megakaryocyte progenitors (CFU-GEMM); and granulocytes and macrophages (CFU-GM). All the cultures were done in duplicate. Data as presented are mean ± SEM. Three to eight independent specimens were analyzed.

### Proliferation Index

Proliferation index was measured by counting the number of pHH3-immunoreactive cells, divided by the total cell number for each section analyzed. Eight random fields were counted for an average number of 500 cells per field. Four specimens per genotype were tested. The primary antibody used was a rabbit monoclonal antibody against pHH3 (1/200 dilution; Cell Signaling, Danvers, MA, United States). The biotinylated secondary antibody was a goat anti-rabbit (1/250; Vector Laboratories, Burlington, ON, Canada).

### Erythrocyte Half-Life

The erythrocyte half-life was evaluated using biotinylation of entire blood cells and monitoring for cell replacement (Gifford et al., 2006). Biotinylation of blood cell was carried out by intravenous injection of 150 μL of 3.25 mg/mL of N-hydroxysulfosuccinimide (NHS) esters (EZ-Link NHS-Biotin reagents; Thermo Fisher Scientific, Saint-Laurent, QC, Canada) for 2 consecutive days. RBCs (3 × 10^6^) obtained from 1 to 5 μL of medial saphenous vein blood were labeled with 1 μg de streptavidin-AlexaFluor 647 (Thermo Fisher Scientific, cat. no. S21374) in 1 mL of PBS. The number of biotinylated cells in circulating blood was monitored at regular intervals by flow cytometry.

### Phenylhydrazine Stress Test and Response to Erythropoietin Induction

Two- to four-months-old mice were injected subcutaneously with 80 mg/kg phenylhydrazine (PHZ) hydrochloride solution in PBS at day 0 and 40 mg/kg at day 1. Erythropoietin (EPO) induction was done by subcutaneous injection of 50 U of human recombinant EPO (Eprex) at day 0. Blood was obtained via the medial saphenous vein at regular intervals for hematocrit and reticulocyte count measurements by flow cytometry after thiazole orange staining as described by Siekmeier et al. (2000). Data are presented as mean ± SEM, and they are the result of four to five independent specimens analyzed.

### Statistical Analyses

Samples were statistically compared with either the analysis of variance (ANOVA) on linear models with Kruskal–Wallis multiple-comparisons test or mixed-effects analysis with Tukey multiple-comparisons test, when appropriate. In the boxplots, the whiskers go down to the smallest value and up to the largest, whereas the boxes extend from the 25th to 75th percentiles with the median. *P* < 0.05 was considered statistically significant.

## Results

### Loss of *Mek* Function in Hematopoietic Cell Lineages Causes Severe Anemia and Neonatal Death

**FIGURE 1:**
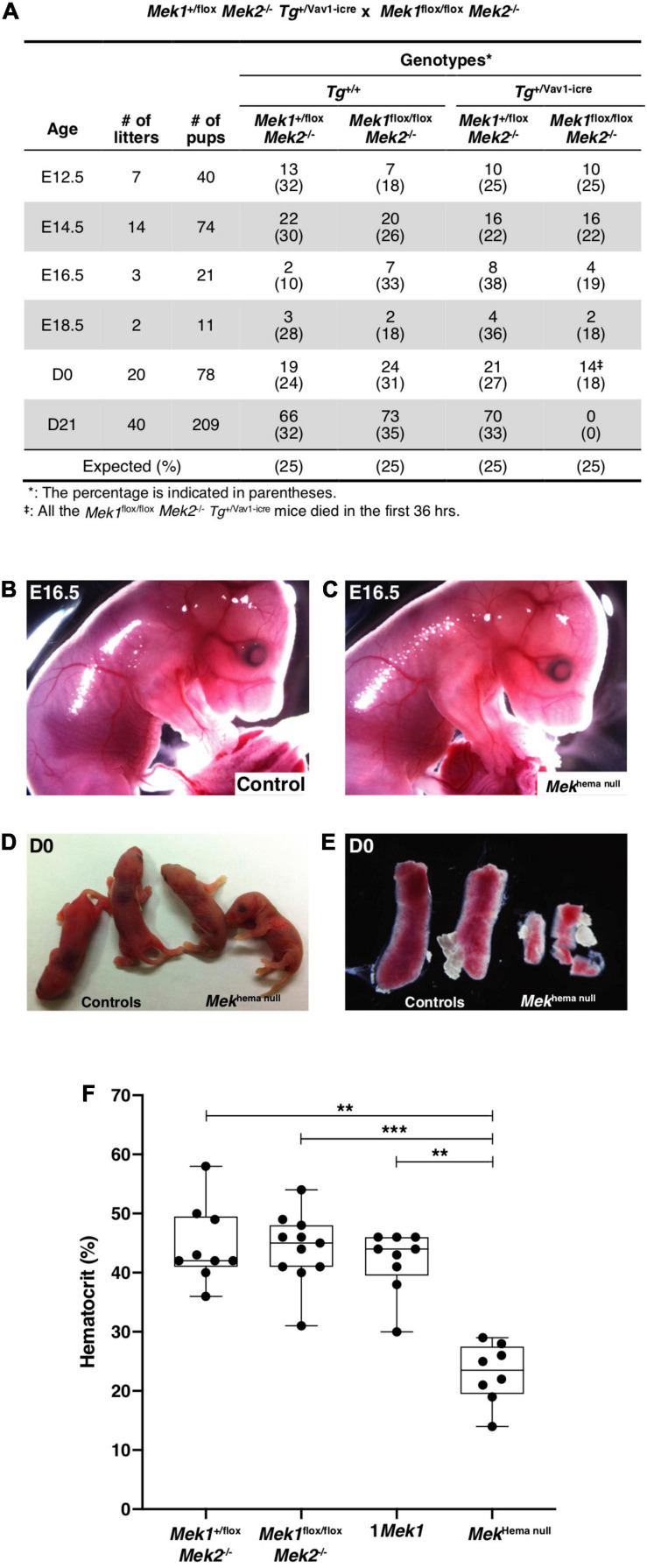
Impact of the conditional *Mek1* deletion with the *Vav1-icre* in the *Mek2*^–/–^ background. **(A)** Ratios of embryos and mice genotypes at different embryonic and adult stages obtained from matings between *Mek1*^+/flox^ *Mek2*^–/–^ *Tg*^+/Vav1–*iCre*^ male with *Mek1*^*flox/flox*^ *Mek*2^–/–^ female. **(B–D)** Gross morphology of E16.5 **(B,C)** and D0 **(D)** control and *Mek*^*hema null*^ animals. Normal vascularization and blood circulation were seen in controls and *Mek*^*hema null*^ embryos at E16.5. **(E)** Macroscopic views showing the hypoplastic *Mek*^*hema null*^ spleen phenotype at D0 compared with spleen from littermate controls. **(F)** Hematocrit at birth. No sign of cyanosis was detected at birth in *Mek*^*hema null*^ embryos, but they died in the first 36 h with a severe anemia. The results are presented as whiskers-and-boxes graph, and each dot represents an individual mouse. ANOVA with Kruskal–Wallis multiple-comparisons test was performed. **P < 0.01, ***P < 0.001.

The specific ablation of *Mek1* function in hematopoietic cell lineages was generated with the *Vav1-icre* deleter mouse line in a *Mek2* null background (de Boer et al., 2003). *Mek1*^+/flox^ *Mek2*^–/–^ *Tg*^+/Vav1–*iCre*^ (thereafter named 1*Mek1*) males were bred with *Mek1*^*flox/flox*^ *Mek*2^–/–^ females to generate *Mek1*^*flox/flox*^ *Mek2*^–/–^ *Tg*^+/Vav1–*icre*^ mutants (*Mek*^*hema null*^). Expected Mendelian ratios were obtained during embryogenesis and up to birth (Figure 1A). At embryonic day (E) 16.5, *Mek*^*hema null*^ mutants appeared phenotypically normal with no obvious defect in RBC production and vasculature (Figures 1B,C). At birth (D0), *Mek*^*hema null*^ newborns looked similar to their control littermates with no sign of cyanosis (Figure 1D). However, all *Mek*^*hema null*^ pups died within the first 36 h of life exhibiting a two-time reduction of hematocrit and a severe decrease in spleen size, indicating anemia and problematic splenic erythropoiesis (Figures 1A,E,F).

**FIGURE 2:**
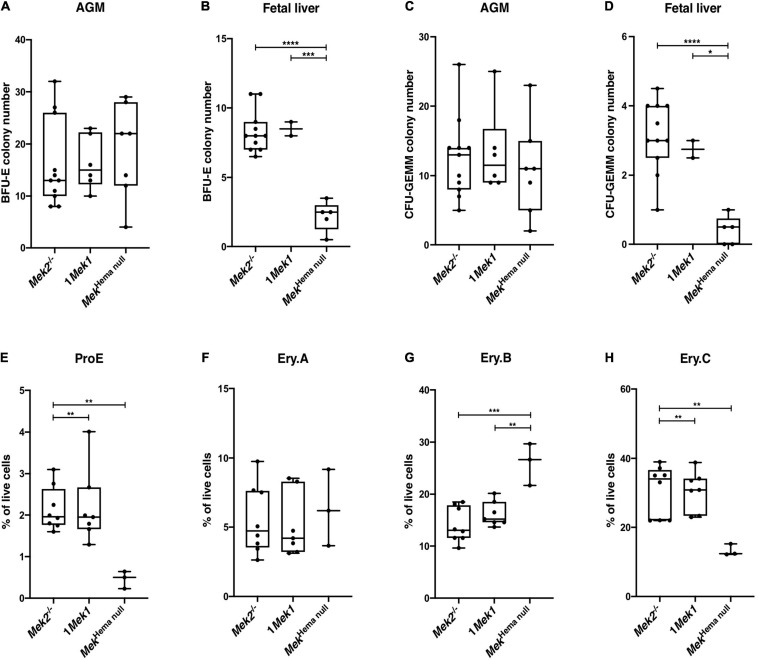
Erythroid progenitors and subsets in *Mek*^*hema null*^ mutants. **(A–F)** Numbers of BFU-E **(A–C)** and CFU-GEMM **(D–F)** colonies obtained after *in vitro* growth of E10.5 AGM region **(A,C)** and E14.5 fetal liver cells **(B,D)** from controls and *Mek*^*hema null*^ embryos are presented. **(E–H)** Erythroid subset analysis by flow cytometry analysis in newborn spleen. Frequency of Ter119^*med*^CD71^*high*^FSC^*high*^ (ProE; **E**), Ter119^*high*^CD71^*high*^FSC^*high*^ (Ery.A; **F**), Ter119^*high*^CD71^*high*^FSC^*low*^ (Ery.B; **G**), and Ter119^*high*^CD71^*low*^FSC^*low*^ (Ery.C; **H**) are presented. They revealed a decrease of the ProE and Ery.B populations and an increase of the Ery.C population in *Mek*^*hema null*^ samples. The results are presented as whiskers-and-boxes graph, and each dot represents an individual mouse. ANOVA with Kruskal–Wallis multiple-comparisons test was performed. *P < 0.05, **P < 0.01, ***P < 0.001, ****P < 0.0001.

Erythropoiesis occurs sequentially at different sites during embryogenesis. It starts in the yolk sac from E7.5 to E10.5, to migrate into the AGM region between E8.5 and E11.5, and then to the placenta from E10.5 to E13.5, and to the fetal liver at E10.5 until birth (Orkin and Zon, 2008). In adult mice, erythropoiesis takes place mainly in bone marrow, but it can also occur in the spleen in response to anemic stress (Paulson et al., 2011). To determine whether anemia and the reduced spleen size seen in *Mek*^*hema null*^ pups were due to defective expansion or differentiation of specific hematopoietic progenitors, we measured the number of colonies of BFU-E and CFU-GEMM by performing methylcellulose colony-forming assays with AGM at E10.5 and fetal liver at E14.5 from control (*Mek2*^–/–^ and 1*Mek1*) and *Mek*^*hema null*^ embryos (Figure 2). No statistical difference was observed in the numbers of BFU-E and CFU-GEMM progenitor populations from AGM of control and *Mek*^*hema null*^ mutants. This result was not surprising since the onset of the *Vav1-iCre* activity occurs around E11.5. However, it precludes the investigation of the role for the ERK pathway at the early stage of hematopoiesis (Figures 2A,C; Bustelo et al., 1993). In contrast, the number of BFU-E colonies from the fetal liver was reduced in *Mek*^*hema null*^ mutants, and only few CFU-GEMM colonies were recovered compared to controls (Figures 2B,D). Thus, the lack of ERK signaling in *Mek*^*hema null*^ mutants perturbed hematopoiesis by affecting the production or the survival of erythroid progenitors, which most likely contributed to the anemia observed in *Mek*^*hema null*^ newborns.

To determine if these embryonic defects translate into abnormal erythropoietic cell differentiation at birth, flow cytometry analyses for the expression of Ter119 and CD71 markers and the forward scatter (FSC) parameter were performed in spleen from newborns to monitor the development of the erythropoietic compartment from proerythroblasts (ProE; Ter119^*med*^CD71^*high*^FSC^*high*^) to successive differentiation stages: basophilic (Ery.A; Ter119^*high*^CD71^*high*^FSC^*high*^), late basophilic and polychromatic (Ery.B; Ter119^*high*^ CD71^*high*^FSC^*low*^), and finally orthochromatic erythroblasts (Ery.C; Ter119^*high*^CD71^*low*^FSC^*low*^ (Guihard et al., 2010; Koulnis et al., 2011). The proportion of committed ProE was reduced by four times in *Mek*^*hema null*^ mutants when compared to *Mek2*^–/–^ and 1*Mek1* control littermates (Figure 2E). Moreover, the proportion of differentiated orthochromatic erythroblasts was decreased by half in *Mek*^*hema null*^ mutants, whereas the proportion of late basophilic and polychromatic erythroblasts was doubled (Figures 2G,H). No change was observed for the basophilic erythroblasts (Figure 2F). These results, combined with the reduced size of the spleen at birth (Figure 1E), suggested a key role for the ERK pathway in erythroid cell commitment and differentiation. In absence of ERK signaling through the loss of both *Mek* genes, reduced proerythroblast commitment and a block at the last steps of erythroid cell maturation occurred.

**FIGURE 3:**
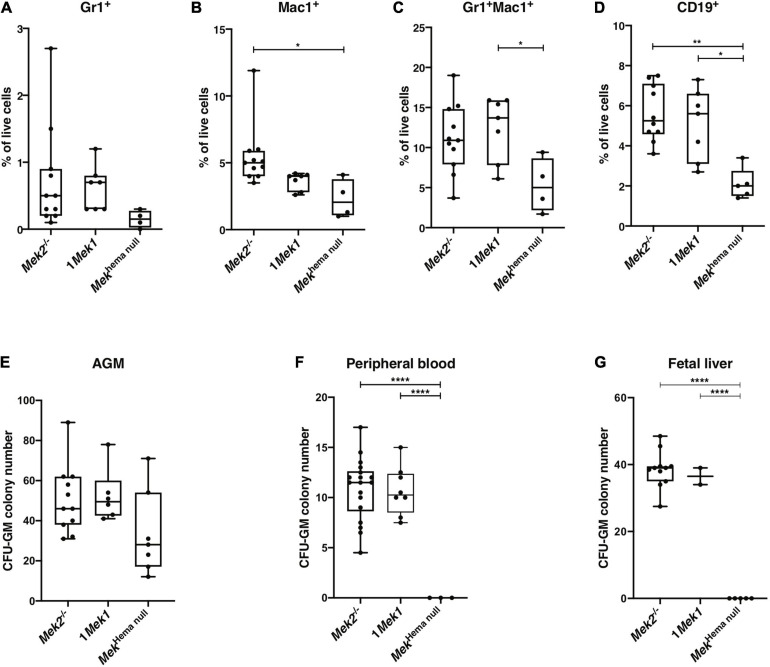
Hematopoiesis cell populations affected in *Mek*^*hema null*^ mutants. **(A–D)** Flow cytometry analyses of newborn spleen revealed a decreased in the proportion of Gr-1^+^ **(A)**, Mac1^+^ **(B)**, Mac1^+^Gr-1^+^ **(C)**, and CD19^+^ **(D)** cells in *Mek*^*hema null*^ mutants compared to control animals. **(E–G)** Numbers of CFU-GM colonies obtained after *in vitro* growth of E10.5 AGM region **(E)**, E12.5 peripheral blood cells **(F)**, and E14.5 fetal liver cells **(G)** from controls and *Mek*^*hema null*^ embryos are presented. A gradual loss of erythroid and myeloid cell progenitors occurred in *Mek*^*hema null*^ embryos during gestation. The results are presented as whiskers-and-boxes graph, and each dot represents an individual mouse. ANOVA with Kruskal–Wallis multiple-comparisons test was performed. *P < 0.05, **P < 0.01, ****P < 0.0001.

As the lack of MEK function was associated with decreased CFU-GEMM, we also analyzed the myeloid differentiation in splenocytes from newborns by flow cytometry. The proportion of Mac1^+^ (also known as CD11b) single-positive and Gr1^+^Mac1^+^ double-positive myeloid cell populations, as well as CD19^+^ B cell populations, was significantly reduced in *Mek*^*hema null*^ mutants, indicating a diminution in monocytes, macrophages, and B cells (Figures 3A–D). Moreover, no statistical differences in granulocyte and macrophage progenitors (CFU-GM) colony numbers, as assessed by methyl cellulose colony formation assay, were detected in AGM at E10.5 (Figure 3E). In contrast, the number of CFU-GM was reduced in peripheral blood at E12.5 and in fetal liver at E14.5 from *Mek*^*hema null*^ embryos when compared to controls (Figures 3F,G). As the size of the spleen in *Mek*^*hema null*^ newborn mice was greatly reduced, the decreased numbers of progenitor cell populations might be even more pronounced supporting the importance of the role of MEK in erythroid and myeloid hematopoietic lineage commitment.

### Mild Anemia in 1*Mek1* and 1*Mek2* Mutants

**FIGURE 4:**
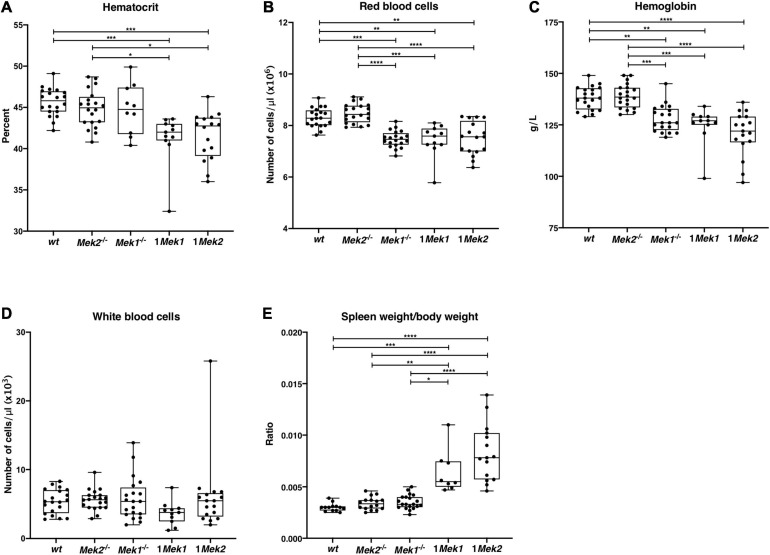
1*Mek1* and 1*Mek2* mutants present a splenomegaly and a trend to anemia. Measurement of the hematologic parameters was performed on *wt*, *Mek2*^–/–^, *Mek1*^–/–^, 1*Mek1*, and 1*Mek2* mutant mice at 2–4 months of age. Hematocrit **(A)**, red blood cell count **(B)**, hemoglobin concentration **(C)**, and white blood cell count **(D)** are presented. **(E)** The spleen weight–to–body weight ratio was significantly reduced in 1*Mek1* and 1*Mek2* compared to controls, indicating splenomegaly. The results are presented as whiskers-and-boxes graph, and each dot represents an individual mouse. ANOVA with Kruskal–Wallis multiple-comparisons test was performed. *P < 0.05, **P < 0.01, ***P < 0.001, ****P < 0.0001.

To tear apart the impact of MEK dosage versus MEK isoform on hematopoiesis, we studied mouse models bearing only one allele of *Mek1* (*Mek1*^+/flox^ *Mek2*^–/–^ *Tg*^+/Vav1–*iCre*^ thereafter named 1*Mek1*) or *Mek2* (*Mek1*^*flox/flox*^ *Mek2*^+/–^ *Tg*^+/Vav1–*iCre*^, 1*Mek2*) in the hematopoietic cell lineages. Both mutant mice were viable, and their hematopoietic parameters were studied from 2 to 4 months of age and compared to samples from *wt* and single *Mek* null mutants [*Mek1*^*flox/flox*^ *Tg*^+/Sox2–*cre*^ (*Mek1*^–/–^) and *Mek2*^–/–^ mice]. While *wt* and *Mek2*^–/–^ mutants presented no difference in hematocrit, hemoglobin concentration, and RBC counts, *Mek1*^–/–^ mutants display significantly reduced hemoglobin concentration and RBC numbers indicating that *Mek1* or *Mek2* genes are not totally interchangeable for these processes (Figures 4A–C). Moreover, reducing *Mek* genes to one allele led to reduction in hematocrit levels, hemoglobin concentration, and RBC numbers in both 1*Mek1* and 1*Mek2* mutants. Of note, the number of circulating white blood cells was similar to *wt* counts in all *Mek* mutants, suggesting that one allele of *Mek* is sufficient to sustain normal leukopoiesis (Figure 4D). Surprisingly, the spleen weight/body weight ratio was two and three times higher in 1*Mek1* and 1*Mek2* mutants, respectively, when compared to controls (Figure 4E).

### Normal Response to Erythropoietic Stress in 1*Mek1* and 1*Mek2* Mutants

**FIGURE 5:**
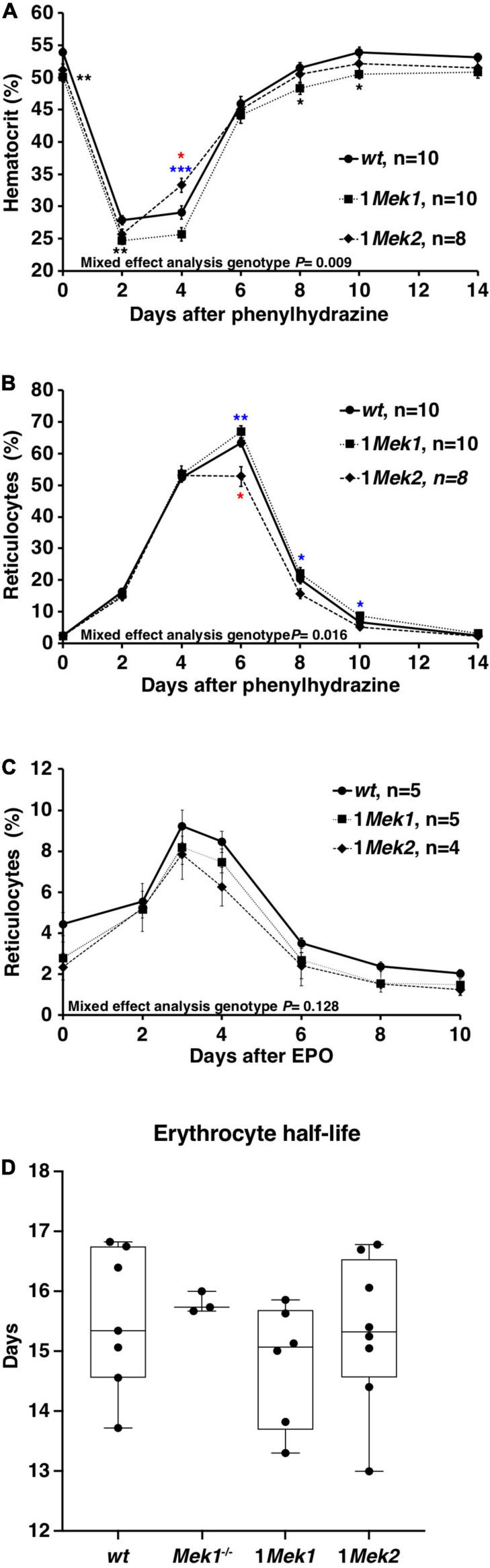
Normal erythropoiesis in 1*Mek1* and 1*Mek2*. **(A,B)** *wt*, 1*Mek1*, and 1*Mek2* mutant mice were injected with PHZ on days 0 and 1. Hematocrit **(A)** and reticulocyte count **(B)** were assessed every 2 days for 2 weeks. A normal response to this erythropoietic stress was observed in mutants. **(C)** The effect of a single subcutaneous injection of EPO on reticulocyte counts was monitored in controls and 1*Mek1* and 1*Mek2* mutants. Blood samples were collected every 2 days for reticulocyte count by flow cytometry. Response to EPO was unchanged in mutants. **(D)** Biotin-reactive agent was injected in tail vein to label RBCs and monitor their survival. The disappearance rate of biotinylated RBCs with time was used to calculate the RBC half-life. No statistical difference was observed between controls and 1*Mek1* and 1*Mek2* mutants. Boxplots depict data distribution; each dot represents one individual. Data are presented as the mean ± SEM **(A–C)**. Mixed-effects analysis with Tukey multiple-comparisons test **(A–C)** and ANOVA **(D)** was performed. *P < 0.05, **P < 0.01, ***P < 0.001. Black: *wt* versus 1*Mek1*; red: *wt* versus 1*Mek1*; blue: 1*Mek1* versus 1*Mek2*.

Fewer circulating RBCs could be explained either by a defective generation or by a decrease in erythrocyte survival. Reduction in ERK signaling may impair the ability of erythropoietic progenitors to correctly respond to proliferative and differentiation signals. This was directly addressed *in vivo* by testing the ability of 1*Mek1* and 1*Mek2* mutants to recover from a hemolytic anemia induced by PHZ (Paulson et al., 2011). PHZ reacts with hemoglobin leading to its degradation, to the lysis of erythrocytes, and to a decreased hematocrit inducing an anemic condition. After injection of PHZ, hematocrit and reticulocyte counts were monitored over a period of 14 days. Hematocrit dropped sharply to around 25% 2 days after PHZ treatment in *wt*, 1*Mek1*, and 1*Mek2* mice (Figure 5A). After this initial drop, hematocrit levels increased. 1*Mek2* mutants showed an accelerated response at day 4, whereas 1*Mek1*mutants presented a delay in recovery. However, all genotypes returned to normal levels after 14 days. The proportion of peripheral blood reticulocytes was also evaluated by flow cytometry and used as an indicator of the erythropoietic proliferation response (Figure 5B). The basal reticulocyte count was less than 2.5% in *wt*, 1*Mek1*, and 1*Mek2* animals. After PHZ treatment, erythropoiesis was induced, and reticulocyte counts increased for all genotypes to reach maximal levels at day 6. The reticulocyte counts were slightly higher in 1*Mek2* mutants at day 6 and lower in 1*Mek1* mutants between days 6 and 10. No significant difference was observed at day 14 between *wt*, 1*Mek1*, and 1*Mek2* animals.

Another approach to test whether proliferation response during erythropoiesis was affected in 1*Mek1* and 1*Mek2* mutants was to treat mice with EPO and to assess if it causes an increase in reticulocyte cell counts. One subcutaneous injection of 50 U of EPO was performed, and reticulocyte counts were monitored by flow cytometry over a period of 10 days. *wt*, 1*Mek1*, and 1*Mek2* mice showed a similar response indicating that the reaction to EPO was not significantly modified in 1*Mek1* and 1*Mek2* mutants versus *wt* animals (Figure 5C). Therefore, no major difference in the hematologic responses to PHZ and EPO was observed in 1*Mek1* and 1*Mek2* mutants that could explain the anemia observed in these mutants.

Alternatively, fewer circulating RBCs can be explained by a decrease in erythrocyte survival. To assess the half-life of circulating RBCs in 2- to 4-month-old 1*Mek1* and 1*Mek2* mutants, we measured the turnover of biotin-labeled RBCs by flow cytometry (Figure 5D). 1*Mek1* and 1*Mek2* RBCs showed a half-life similar to that of *wt* and *Mek1*^–/–^ mutants indicating that RBC survival was normal.

### Hematopoietic Stem and Progenitor Cells Are Altered in 1*Mek1* Mutants

**FIGURE 6:**
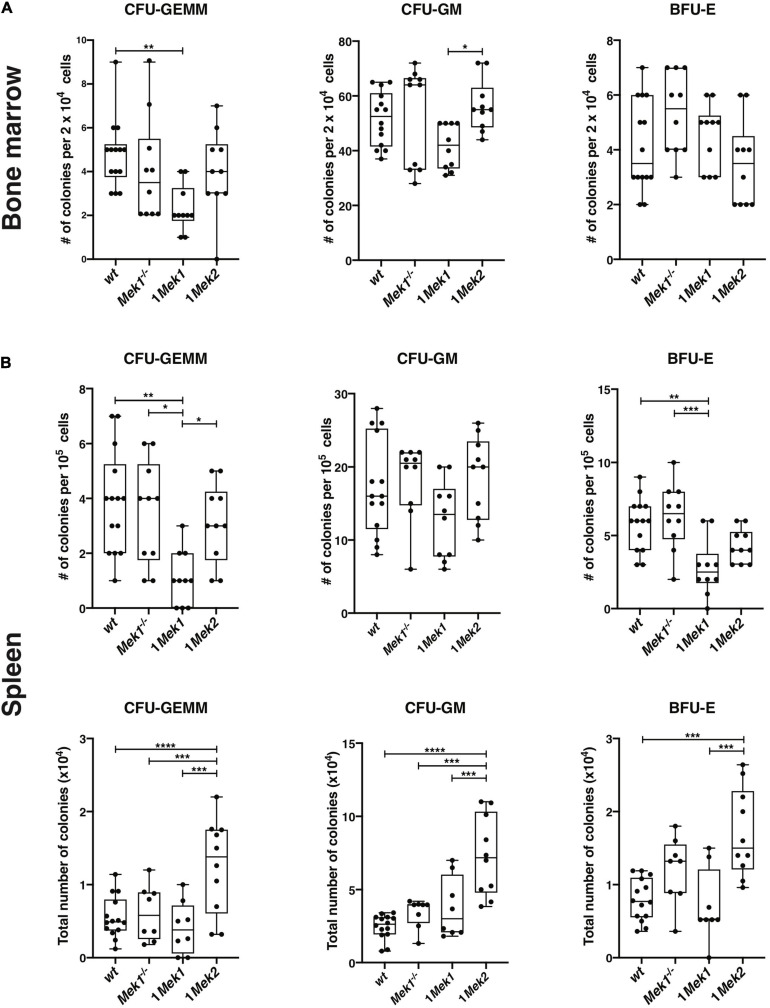
Counts of hematopoietic progenitors in bone marrow and spleen from 1*Mek1* and 1*Mek2* mutants. CFU-GEMM, CFU-GM, and BFU-E from *wt* and *Mek* mutant bone marrow **(A)** and spleen **(B)** were measured after 7 days in methylcellulose with cytokines. A decrease in the CFU-GEMM and CFU-GM populations was observed in bone marrow of 1*Mek1* mutants, whereas an increase in the total number of progenitor populations was detected in spleen from 1*Mek2* mutants. The results are presented as whiskers-and-boxes graph, and each dot represents an individual mouse. ANOVA with Kruskal–Wallis multiple-comparisons test was performed. *P < 0.05, **P < 0.01, ***P < 0.001, ****P < 0.0001.

To verify whether the anemia in 1*Mek1* and 1*Mek2* resulted from a defect in a specific hematopoietic progenitor population, colony-forming assays in methylcellulose were performed with bone marrow and spleen from 10-week-old *wt*, *Mek1*^–/–^, 1*Mek1*, and 1*Mek2* mice. While *Mek1*^–/–^ and 1*Mek2* mice behaved like *wt* controls in bone marrow, 1*Mek1* mice displayed altered early hematopoiesis. Indeed, in 1*Mek1* mutants, the numbers of multipotent CFU-GEMM and myeloid CFU-GM progenitors were significantly decreased (Figure 6A).

**FIGURE 7:**
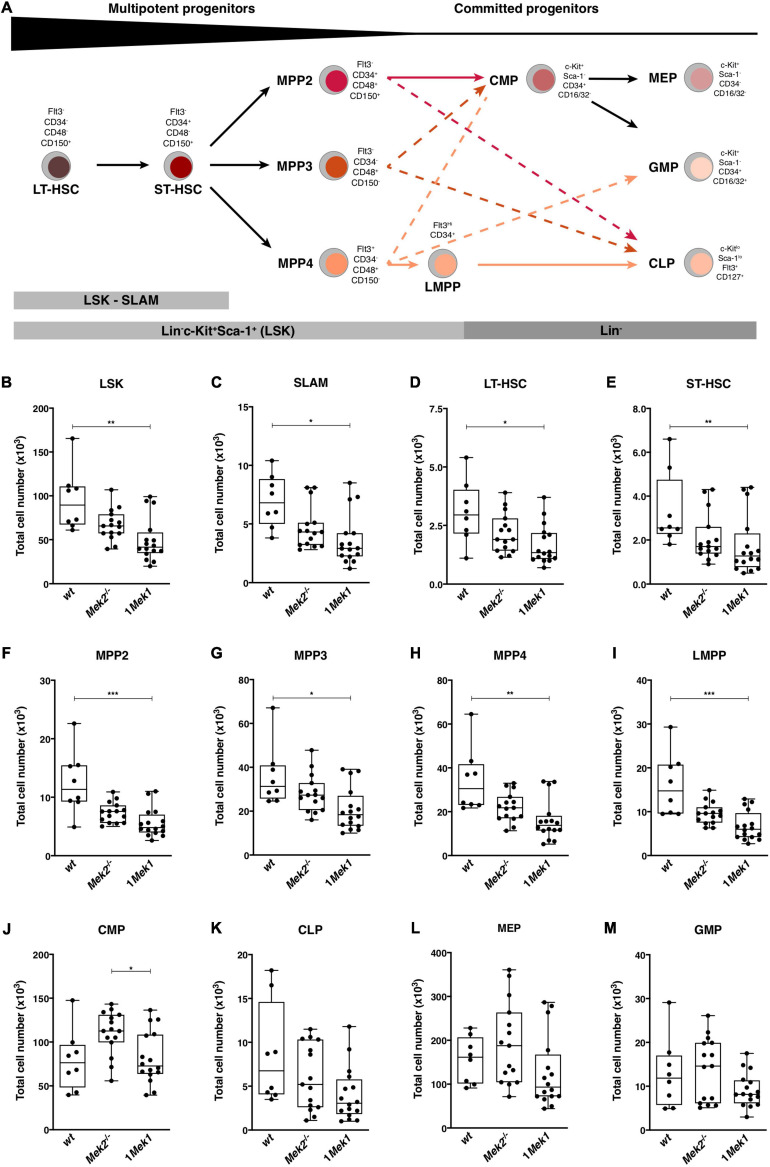
Reduced numbers of HSCs and multipotent HSC progenitors in bone marrow from 1*Mek1* mutants. **(A)** Schematic representation of lineage determination in the mouse hematopoietic system with the flow cytometric gating strategy used to monitor the major classes of mouse hematopoietic stem and progenitor cells. **(B–M)** 1*Mek1* mutant mice displayed a cell autonomous reduction in multipotent progenitors and lymphoid-biased progenitors in the bone marrow. Absolute numbers of LSK (**B**, Lin^–^c-Kit^+^Sca-1^+^), SLAM (**C**, Lin^–^c-Kit^+^Sca-1^+^CD48^–^CD150^+^), LT-HSC (**D**, Lin^–^c-Kit^+^Sca-1^+^CD48^–^CD150^+^Flt3^–^CD34^–^), ST-HSC (**E**, Lin^–^c-Kit^+^Sca-1^+^CD48^–^CD150^–^Flt3^–^CD34^–^), MPP2 (**F**, Lin^–^Sca-1^+^c-Kit^+^Flt3^–^CD34^–^CD48^+^CD150^+^), MPP3 (**G**, Lin^–^Sca-1^+^c-Kit^+^Flt3^–^CD34^–^CD48^+^ CD150^–^), MPP4 (**H**, Lin^–^Sca-1^+^c-Kit^+^Flt3^+^CD34^–^CD48^+^CD150^–^), LMPP (**I**, Lin^–^c-Kit^+^Sca-1^+^ Flt3^*high*^CD34^+^), CMP (**J**, Lin^–^c-Kit^+^Sca-1^–^CD34^+^CD16/32^–^), CLP (**K**, Lin^–^c-Kit^*low*^Sca-1^*low*^Flt3^+^CD127^+^), MEP (**L**, Lin^–^c-Kit^+^Sca-1^+^CD34^–^CD16/32^–^), and GMP (**M**, Lin^–^c-Kit^+^Sca-1^–^CD34^+^CD16/32^+^) in bone marrow of *wt*, *Mek2*^–/–^, and 1*Mek1* mutants are presented. Data are from at least four independent experiments. The results are presented as whiskers-and-boxes graph, and each dot represents an individual mouse. ANOVA with Kruskal–Wallis multiple-comparisons test was performed. *P < 0.05, **P < 0.01, ***P < 0.001.

HSCs generate mature leukocytes through successive differentiation steps of increasingly committed HSC progenitors (Freitas et al., 2017; Zhang et al., 2018; Singh and Cancelas, 2020). To determine whether impaired HSC maturation contributed to the 1*Mek1* phenotype, global hematopoietic development was assessed in controls and 1*Mek1* mice following the gating strategy described in Supplementary Figure 1 (Freitas et al., 2017). Flow cytometry analyses with the appropriate combination of cell markers were performed on bone marrow and spleen from 10-week-old mice to resolve each progenitor population (Figure 7A). Bone marrow from 1*Mek1* mutants showed a two times reduction in the number of immature HSC between stages LT-HSC and LMPP when compared to *wt* specimens (Figures 7B–I). A trend for a decrease was also observed for the common lymphoid progenitor (CLP) population (Figure 7K), whereas no difference was measured for common myeloid progenitors (CMPs; Figure 7J), megakaryocyte-erythroid progenitors (MEPs; Figure 7L), and granulocyte–macrophage progenitors (GMPs; Figure 7M). Thus, in bone marrow from 1*Mek1* mutants, multipotent progenitors were less abundant, but the number of committed progenitors recovered at later stages indicated that one functional *Mek1* allele was sufficient to maintain hematopoietic progenitor homeostasis in accordance with the normal levels of mature myeloid cells observed (Figure 3).

In the spleen, the proportions of CFU-GEMM and BFU-E progenitors were reduced in 1*Mek1* mutants, but their total numbers in the organ were not significantly diminished compared to controls. Moreover, spleen from 1*Mek1* mutants presented no significant change in HSC maturation when compared to *wt* and *Mek2*^–/–^ controls (Supplementary Figure 2). In contrast, the total numbers of CFU-GM, CFU-GEMM, and BFU-E progenitors were significantly increased in 1*Mek2* mutants, which might contribute to the expansion of the spleen in 1*Mek2* mutants (Figure 6B).

**FIGURE 8:**
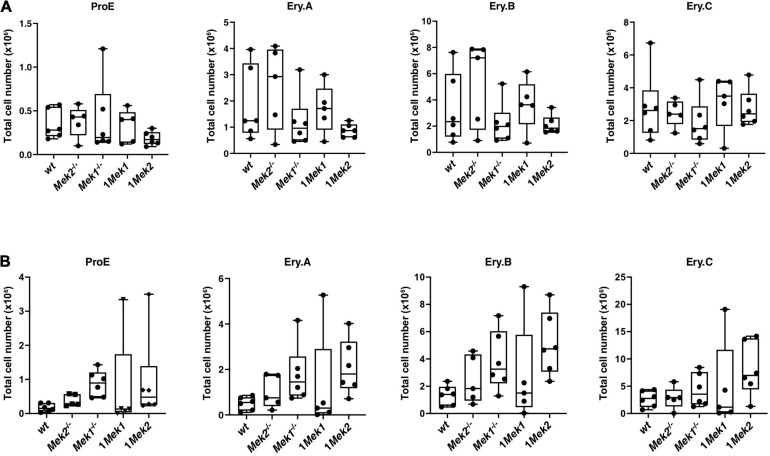
Erythroid subsets in bone marrow and spleen of adult *Mek* mutants. Erythroid subset analysis by flow cytometry on bone marrow **(A)** and spleen **(B)** from *wt* and *Mek* mutant adults. Frequency of Ter119^*med*^CD71^*high*^FSC^*high*^ (ProE), Ter119^*high*^CD71^*high*^FSC^*high*^ (Ery.A), Ter119^*high*^CD71^*high*^FSC^*low*^ (Ery.B), and Ter119^*high*^CD71^*low*^FSC^*low*^ (Ery.C) are presented. The results are presented as whiskers-and-boxes graph, and each dot represents an individual mouse. ANOVA with Tukey multiple-comparisons test was performed. No significant difference was observed.

To determine whether erythropoietic cell differentiation defects were associated with reduced *Mek* function, ProE, Ery.A, Ery.B, and Ery.C cell populations were assessed in bone marrow and spleen from *wt*, *Mek2*^–/–^, *Mek1*^–/–^, 1*Mek1*, and 1*Mek2* adults. No significant difference was observed in bone marrow and spleen (Figure 8). However, Ery.A, Ery.B, and Ery.C cell populations showed a tendency to increase in 1*Mek2* mutant spleens, suggesting that a distinct differentiation response may exist between 1*Mek1* and 1*Mek2* mutants (Figure 8B).

**FIGURE 9:**
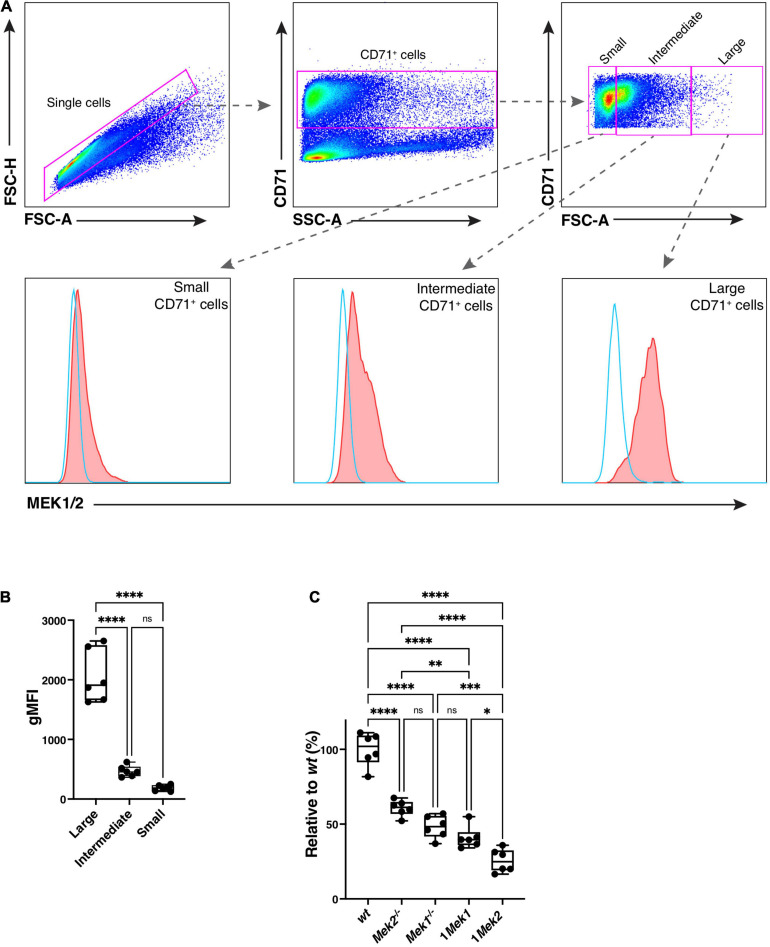
Loss of MEK Protein Expression During the Erythropoietic Cell Differentiation. **(A)** Representative dot plots show the flow cytometry gating strategy used to quantitate the levels of MEK1 and MEK2 proteins in CD71^+^ erythroid precursors isolated from bone marrow of *wt* and *Mek* mutants. MEK protein levels in *wt* specimens were measured in CD71^+^ cells of different sizes using the forward scatter. The larger cells correspond to the ProE and Ery.A cells, whereas the cells with the intermediate and small sizes correspond to more differentiated Ery.B cells. **(B)** The geometric mean of the fluorescence intensity (gMFI) for intracellular staining of MEK1/2 was used to quantify the levels of MEK protein. High levels of MEK protein were detected in ProE and Ery.A precursors (no antibody in blue and with anti-MEK1/2 in red). **(C)** Quantification of MEK protein levels in the allelic series of *Mek* mutants relative to the levels in *wt* was performed for the large CD71^+^ cells. *P < 0.05, **P < 0.01, ***P < 0.001, ****P < 0.0001.

The impact of MEK dosage versus MEK isoform on the erythroid phenotype was assessed by quantifying the levels of MEK proteins by flow cytometry in CD71^+^ erythroid precursors using an antibody that cross-reacts with MEK1 and MEK2 (Figure 9A; Aoidi et al., 2016). Cell size was determined by the forward scatter: the larger cells correspond to ProE and Ery.A cell populations, whereas cells with intermediate and small sizes correspond to the more differentiated Ery.B cells. High levels of MEK proteins were detected in ProE and Ery.A erythroid precursors from *wt* specimens, whereas 5–10 times lower levels of MEK protein were measured in the more differentiated Ery.B erythroid populations (Figure 9B). Quantification of MEK protein levels in the allelic series of *Mek* mutants was performed for ProE and Ery.A erythroid precursors. Levels of MEK protein were maximal in *wt* specimens, and they gradually decreased in *Mek2*^–/–^, *Mek1*^–/–^, and 1*Mek1*, to finally be the lowest in 1*Mek2* mutants (Figure 9C). The similar levels of MEK observed in *Mek1*^–/–^ (two functional alleles of *Mek2*) and 1*Mek1* (one functional allele of *Mek1*) specimens and the lowest MEK levels in 1*Mek2* (one functional allele of *Mek2*) established that the *Mek1* allele generates roughly twice the amount of MEK protein than the *Mek2* allele. This suggests that MEK dosage rather than MEK isoform might be responsible for the different phenotypes.

## Discussion

Our previous characterization of the *Mek1 Mek2* compound mutant mice has provided genetic evidence establishing the crucial and unique roles played by MEK1 and MEK2 in signal transduction during development. Using a *Mek1* conditional allele and lineage-specific *Cre* mouse lines in a *Mek2* null background, we showed that deletion of both *Mek1* and *Mek2* genes is necessary to generate phenotypes during gliogenesis, respiratory track development, skin formation, kidney branching, and male gonad ontogeny, revealing MEK functional redundancy in several embryonic processes (Scholl et al., 2007; Newbern et al., 2008; Yamashita et al., 2011; Li et al., 2012; Boucherat et al., 2014; Ihermann-Hella et al., 2014; Boucherat et al., 2017). During placenta formation, MEK1 and MEK2 can also substitute for each other, but a minimum threshold of MEK proteins is required for the proper development of extraembryonic structures and embryo survival (Nadeau and Charron, 2014; Aoidi et al., 2016). Here, we showed that the complete absence of MEK function in hematopoietic cell lineages in *Mek*^*hema null*^ mutants causes neonatal death, confirming the essential role of the ERK pathway in hematopoiesis. Conversely, mice that retained one functional allele of either *Mek1* (1*Mek1*) or *Mek2* (1*Mek2*) gene in hematopoietic lineages can survive until adulthood. This establishes that MEK1 and MEK2 are partly interchangeable during early hematopoiesis as previously shown for other developmental processes.

*Mek*^*hema null*^ mutants died shortly after birth from a severe anemia, even though *Vav1-icre* expression occurs much earlier, around E11.5, in definitive HSCs (dHSCs), which are known to be involved in the generation of the definitive erythroid, myeloid, and lymphoid lineages (de Boer et al., 2003; Medvinsky et al., 2011). In vertebrates, there are two main waves of hematopoiesis. First, the embryonic wave starts around E7.5 in the yolk sac and then migrates into AGM and placenta (Orkin and Zon, 2008). Embryonic hematopoiesis is responsible for the expansion of transient populations of nucleated primitive erythrocytes and some myeloid cells. Second, the definitive hematopoiesis starts at E10.5 in the fetal liver, which then becomes the major hematopoietic organ of the fetus (Bustelo et al., 1993; Ogilvy et al., 1999). Early production of RBCs is essential during development to sustain the rapid growth of the embryo after gastrulation. Our data indicated that embryonic hematopoiesis and few days of definitive hematopoiesis in *Mek*^*hema null*^ mutants are sufficient to sustain the normal development of the embryo until birth. Inactivation of the two alleles of both *Mek1* and *Mek2* genes in dHSCs at E11.5 jeopardized the renewal of erythroid populations contributing to the anemia at birth. The anemia observed in *Mek*^*hema null*^ newborns is most likely the cause of their death. However, the severe reduction in myeloid and lymphoid cell populations seen in the spleen of *Mek*^*hema null*^ newborns raised the possibility that the HSC pools are affected and that a compromised immune system contributes to septicemia causing the death of *Mek*^*hema null*^ mutants. For instance, it was reported that the mutation of the *Spi1* gene, which encodes the PU.1 transcription factor specific to hematopoietic lineages, affects lymphoid development and causes septicemia and death at birth (Mckercher et al., 1996).

Mice carrying only one functional *Mek* allele in hematopoietic cell lineages survived after birth. Characterization of the hematologic parameters of 1*Mek1* and 1*Mek2* mutant mice revealed small but significant reductions of hematocrit, RBC numbers, and hemoglobin concentration, suggesting defective erythropoiesis. In bone marrow, 1*Mek1* mutant mice presented altered early myelopoiesis, whereas 1*Mek2* mutant mice behaved like control animals. In contrast, in the spleen, no significant differences in total hematopoietic progenitors CFU-GEMM, CFU-GM, and BFU-E were detected in 1*Mek1* mutants, whereas 1*Mek2* mutants showed a significant increase (Figure 6B). The 1*Mek2* phenotype is evocative of the increased accumulation of erythroid progenitors in the spleen of *Erk1*^–/–^ mutants (Guihard et al., 2010). In contrast to the augmented erythropoiesis in *Erk1*^–/–^ mutants, 1*Mek2* mutants showed a mild anemia. In fact, both 1*Mek1* and 1*Mek2* mutants presented similar levels of anemia even if 1*Mek2* mice showed higher levels of hematopoietic progenitors and a stronger erythropoietic stress response compared to *wt* and 1*Mek1* animals. However, 1*Mek1* and 1*Mek2* mutants showed normal erythroid precursor populations in bone marrow and spleen at late stages of erythroid differentiation (Figure 8). The exact cause of anemia in 1*Mek1* and 1*Mek2* mutants remains unresolved, but the return to normal levels of erythroid precursors suggests that the defect occurs at early stages of erythropoiesis with recovery later on.

We have previously shown that the *Mek1* gene produces twice the amount of MEK protein and activity than *Mek2*. Analysis of *Mek1 Mek2* allelic series has demonstrated that the severity of the placenta phenotype correlated with the amount of MEK protein and activity independently of the MEK isoform produced (Aoidi et al., 2016). Accordingly, 1*Mek1* animals produce roughly twice the amount of MEK protein than 1*Mek2* mice in early erythroid precursor cell (Figure 9C). A gradient of MEK protein from the highest level to the lowest in the *wt*, *Mek2*^–/–^, *Mek1*^–/–^, 1*Mek1*, and 1*Mek2* was observed. This gradient of MEK protein should lead to a gradient of ERK activation allowing threshold levels of ERK signaling to be attained in 1*Mek1* mice but not in 1*Mek2* mutants. A minimal activity of the ERK pathway might be required to restrain the hematopoietic splenic progenitor expansion. Moreover, the reduction in MEK proteins at late stages of erythroid cell differentiation indicates that the ERK pathway acts mainly at earlier stages.

The reduction in the total numbers of HSC and CFU-GEMM and CFU-GM progenitors was specific to the bone marrow of 1*Mek1* mutants. This phenotype is reminiscent of the HSC homing and maintenance defects previously described in *Erk1*^–/–^ mice, which was attributed to osteopetrosis due to abnormal myeloid lineage progenitors and impaired osteoclastogenesis and osteoclast function (Saulnier et al., 2012). The impact of the 1*Mek1* mutation on bone remodeling, HSC homing, and maintenance remains to be determined to establish if these factors can contribute to the decreased HSC populations in the bone marrow.

Finally, in 1*Mek1* and 1*Mek2* mutants, the erythrocyte half-life and the erythropoiesis response to hemolytic stress and to EPO remained unaffected, suggesting a relatively normal erythropoietic response despite a defective erythroid homeostasis.

In summary, the conditional deletion of *Mek* function in hematopoietic cell lineages unveiled the essential role of the ERK pathway in erythroid cell commitment and differentiation and the lack of ERK signaling causing a block at the last steps of erythroid cell differentiation. The characterization of our allelic series also revealed the specific contribution of each *Mek* gene in erythropoiesis during gestation despite a minimal impact on the process. The splenomegaly observed in mutants cannot be explained by the almost normal number of erythroid committed progenitors and precursors, which suggests that other hematopoietic cell lineages may also be affected in *Mek* mutants.

## Data Availability Statement

The raw data supporting the conclusions of this article will be made available by the authors, without undue reservation.

## Ethics Statement

The animal study was reviewed and approved by the Université Laval’s Animal Protection Committee (CPAUL) and C2EA-26, Animal Care and Use Committee, Villejuif, France.

## Author Contributions

LB, S-PF-B, ME, and JC: conceptualization. LB, S-PF-B, VR, SR, and NH: investigation. JC: writing – original draft. LB, VR, NH, KB, ME, and JC: writing – review and editing. KB, ME, and JC: funding acquisition and resources. ME, KB, and JC: supervision. All authors contributed to the article and approved the submitted version.

## Conflict of Interest

The authors declare that the research was conducted in the absence of any commercial or financial relationships that could be construed as a potential conflict of interest.

## Publisher’s Note

All claims expressed in this article are solely those of the authors and do not necessarily represent those of their affiliated organizations, or those of the publisher, the editors and the reviewers. Any product that may be evaluated in this article, or claim that may be made by its manufacturer, is not guaranteed or endorsed by the publisher.

## Supplementary Material

Supplementary Figure 1 Flow cytometry gating strategy for delineating the major classes of mouse hematopoietic stem and progenitor cells. Representative dot plots show the flow cytometry gating strategy used for LSK (Lin^–^c-Kit^+^Sca-1^+^), SLAM (LSK CD48^–^CD150^+^), LT-HSC (LSK CD48^–^CD150^+^Flt3^–^CD34^–^), ST-HSC (LSK CD48^–^CD150^+^Flt3^–^CD34^+^), MPP2 (LSK Flt3^–^CD48^+^CD150^+^), MPP3 (LSK Flt3^–^CD48^+^CD150^–^), MPP4 (LSK Flt3^+^CD48^+/–^CD150^–^), LMPP (LSK Flt3^*high*^CD34^+^), CMP (LSK CD34^+^CD16/32^–^), CLP (Lin^–^c-Kit^*low*^Sca-1^*low*^Flt3^+^CD127^+^), MEP (Lin^–^c-Kit^+^Sca-1^–^CD34^–^CD16/32^–^), and GMP (Lin^–^c-Kit^+^Sca-1^–^CD34^+^CD16/32^+^) in the mouse bone marrow (BM).

Supplementary Figure 2 Numbers of HSCs and multipotent HSC progenitors in spleen from 1*Mek1* mutants. **(A–L)** Absolute numbers of LSK (**A**, Lin^–^c-Kit^+^Sca-1^+^), SLAM (**B**, Lin^–^c-Kit^+^Sca-1^+^CD48^–^CD150^+^), LT-HSC (**C**, Lin^–^c-Kit^+^Sca-1^+^CD48^–^CD150^+^Flt3^–^CD34^–^), ST-HSC (**D**, Lin^–^c-Kit^+^Sca-1^+^CD48^–^CD150^–^Flt3^–^CD34^–^), MPP2 (**E**, Lin^–^Sca-1^+^c-Kit^+^Flt3^–^CD34^–^CD48^+^CD150^+^), MPP3 (**F**, Lin^–^Sca-1^+^c-Kit^+^Flt3^–^CD34^–^CD48^+^ CD150^–^), MPP4 (**G**, Lin^–^Sca-1^+^c-Kit^+^Flt3^+^CD34^–^CD48^+^CD150^–^), LMPP (**H**, Lin^–^c-Kit^+^Sca-1^+^ Flt3^*high*^CD34^+^), CMP (**I**, Lin^–^c-Kit^+^Sca-1^–^CD34^+^CD16/32^–^), CLP (**J**, Lin^–^c-Kit^*low*^Sca-1^*low*^Flt3^+^CD127^+^), MEP (**K**, Lin^–^c-Kit^+^Sca-1^+^CD34^–^CD16/32^–^), and GMP (**L**, Lin^–^c-Kit^+^Sca-1^–^CD34^+^CD16/32^+^) in spleen of *wt*, *Mek2*^–/–^, and 1*Mek1* mutants are presented. Data are from at least four independent experiments. The results are presented as whiskers-and-boxes graph, and each dot represents an individual mouse. ANOVA with Kruskal–Wallis multiple-comparisons test was performed. ^∗^*P* < 0.05.
