# Supplementary figures and images for: Mek1 and Mek2 Functional Redundancy in Erythropoiesis
Source: Front Cell Dev Biol. 2021 Jul 27;9:639022. doi: 10.3389/fcell.2021.639022 (PMC8353236; doi:10.3389/fcell.2021.639022)

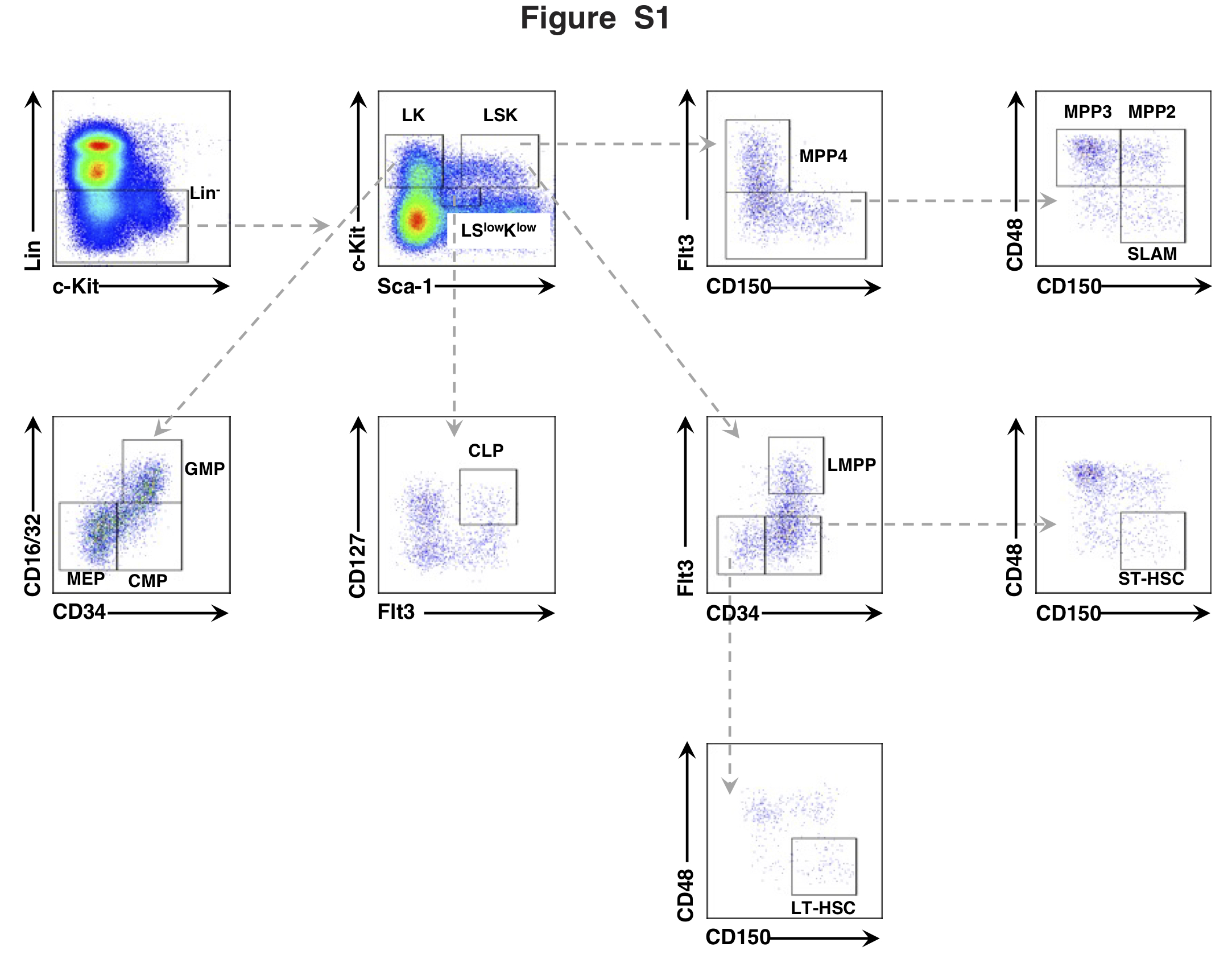

Supplement: Supplementary Figure 1 — Flow cytometry gating strategy for delineating the major classes of mouse hematopoietic stem and progenitor cells. Representative dot plots show the flow cytometry gating strategy used for LSK (Lin–c-Kit+Sca-1+), SLAM (LSK CD48–CD150+), LT-HSC (LSK CD48–CD150+Flt3–CD34–), ST-HSC (LSK CD48–CD150+Flt3–CD34+), MPP2 (LSK Flt3–CD48+CD150+), MPP3 (LSK Flt3–CD48+CD150–), MPP4 (LSK Flt3+CD48+/–CD150–), LMPP (LSK Flt3highCD34+), CMP (LSK CD34+CD16/32–), CLP (Lin–c-KitlowSca-1lowFlt3+CD127+), MEP (Lin–c-Kit+Sca-1–CD34–CD16/32–), and GMP (Lin–c-Kit+Sca-1–CD34+CD16/32+) in the mouse bone marrow (BM). [file Image_1.jpeg]

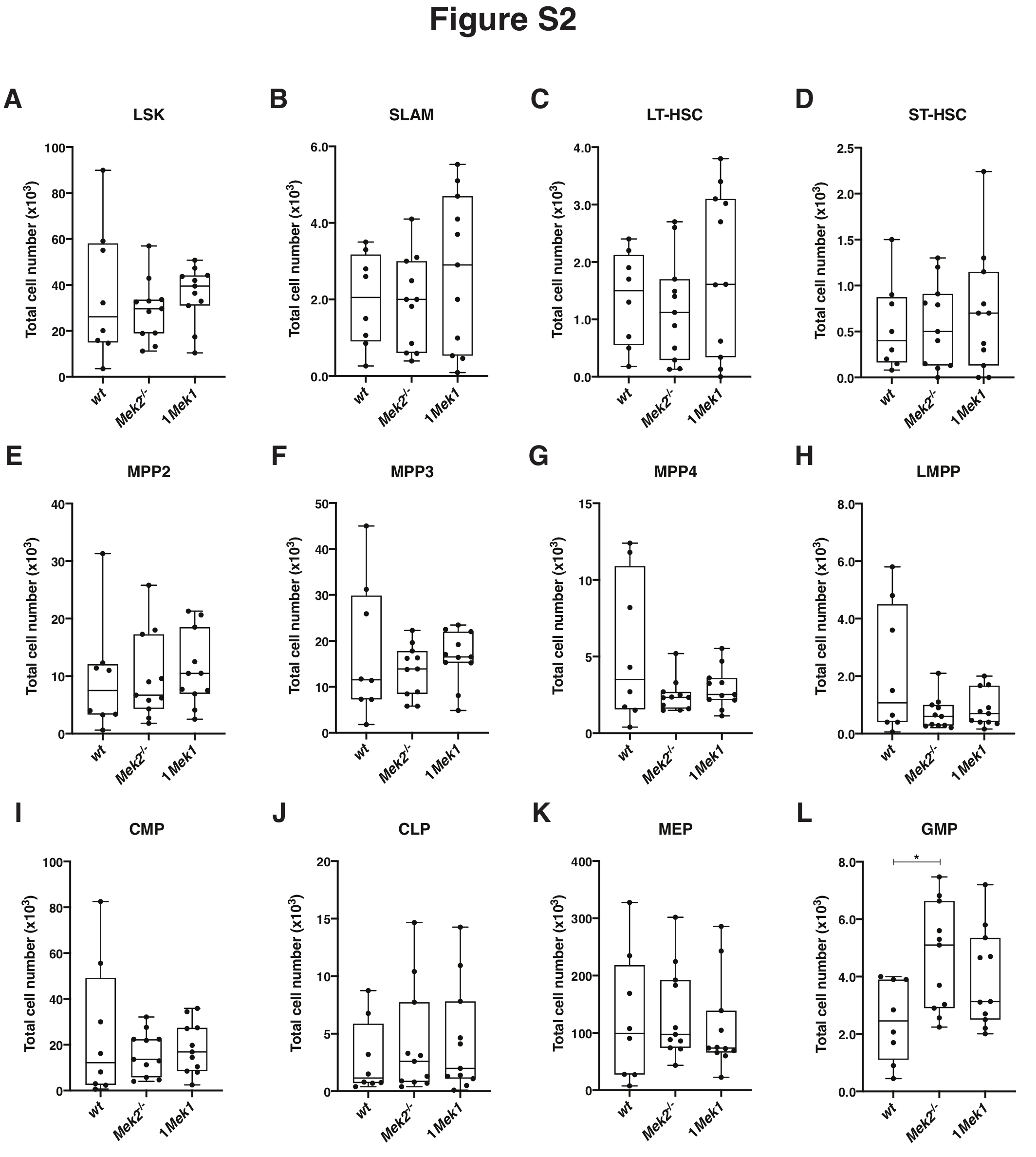

Supplement: Supplementary Figure 2 — Numbers of HSCs and multipotent HSC progenitors in spleen from 1Mek1 mutants. (A–L) Absolute numbers of LSK (A, Lin–c-Kit+Sca-1+), SLAM (B, Lin–c-Kit+Sca-1+CD48–CD150+), LT-HSC (C, Lin–c-Kit+Sca-1+CD48–CD150+Flt3–CD34–), ST-HSC (D, Lin–c-Kit+Sca-1+CD48–CD150–Flt3–CD34–), MPP2 (E, Lin–Sca-1+c-Kit+Flt3–CD34–CD48+CD150+), MPP3 (F, Lin–Sca-1+c-Kit+Flt3–CD34–CD48+ CD150–), MPP4 (G, Lin–Sca-1+c-Kit+Flt3+CD34–CD48+CD150–), LMPP (H, Lin–c-Kit+Sca-1+ Flt3highCD34+), CMP (I, Lin–c-Kit+Sca-1–CD34+CD16/32–), CLP (J, Lin–c-KitlowSca-1lowFlt3+CD127+), MEP (K, Lin–c-Kit+Sca-1+CD34–CD16/32–), and GMP (L, Lin–c-Kit+Sca-1–CD34+CD16/32+) in spleen of wt, Mek2–/–, and 1Mek1 mutants are presented. Data are from at least four independent experiments. The results are presented as whiskers-and-boxes graph, and each dot represents an individual mouse. ANOVA with Kruskal–Wallis multiple-comparisons test was performed. ∗P < 0.05. [file Image_2.jpeg]
